# Psychosocial recovery and nutritional rehabilitation in residential treatment for anorexia nervosa: an exploratory longitudinal multi-frequency bioelectrical impedance analysis study

**DOI:** 10.3389/fpsyt.2026.1883192

**Published:** 2026-09-03

**Authors:** Francesco Monaco, Annarita Vignapiano, Stefania Landi, Rossella Bonifacio, Daniela Falchetta, Emanuela Ferrara, Ernesta Panarello, Bruno Attianese, Annaluce Caputo, Luca Steardo, Gennaro Sosto, Giulio Corrivetti

**Affiliations:** 1 Department of Mental Health ASL Salerno, Salerno, Italy; 2 European Biomedical Research Institute of Salerno, Salerno, Italy; 3 General Management, ASL Salerno, Salerno, Italy

**Keywords:** anorexia nervosa, bioelectrical impedance analysis, body image disturbance, eating disorder psychopathology, exercise dependence, nutritional rehabilitation, psychiatric rehabilitation, residential treatment

## Abstract

**Background:**

Nutritional rehabilitation is widely considered a prerequisite for recovery in anorexia nervosa (AN), given the profound effects of malnutrition on cognitive, emotional, and behavioral functioning. However, whether psychiatric improvement during residential treatment is directly coupled with the degree of nutritional restoration remains insufficiently understood. This study investigated longitudinal changes in body composition and psychosocial outcomes during residential psychiatric rehabilitation for AN, with the aim of informing the evaluation and planning of intensive rehabilitation programmes.

**Methods:**

This exploratory longitudinal observational study included 19 female patients with AN or atypical AN (mean age 20.4 ± 5.6 years) admitted to a specialized residential programme. Multi-frequency bioelectrical impedance analysis (BIA) was performed at admission (T0) and discharge (T1; ≈ 5 months). Diagnoses were confirmed via the Eating Disorder Examination (EDE) and psychiatric comorbidities via the SCID-5. Psychopathology and body image were assessed using the EDE-Q, EDI-3, SCL-90, CIA, EDS-R, and BUT. Wilcoxon signed-rank tests evaluated T0–T1 changes; Spearman correlations assessed nutritional-psychiatric associations. Benjamini-Hochberg correction was applied to all secondary outcomes.

**Results:**

BMI increased significantly (p = 0.042, r = 0.35), whereas FFM and SMM showed non-significant medium-effect-size upward trends. Large and significant improvements were observed in eating disorder psychopathology (EDE-Q: r = 0.74), psychological distress (SCL-90 GSI: r = 0.66), functional impairment (CIA: r = 0.62), exercise dependence (EDS-R: r = 0.72), and body image disturbance (BUT GSI: r = 0.88, the largest effect size in the study). Sixteen of 18 EDI-3 subscales, all nine SCL-90 dimensions, all seven EDS-R subscales, and all seven BUT-A indices improved significantly after false discovery rate correction. Nutritional and psychiatric change scores were not significantly correlated across any pairing.

**Conclusions:**

Five months of residential treatment produced large-magnitude psychiatric improvements alongside modest nutritional gains. The absence of significant somatic-psychiatric correlations should be interpreted with caution: the study had only 37.5% power to detect medium-sized associations (rs = 0.40), yielding a Type II error probability of approximately 62.5%. These findings are hypothesis-generating and do not permit inference about the degree of coupling between nutritional and psychiatric recovery in AN.

## 1 Introduction

Nutritional psychiatry has established that nutritional status is not merely a physiological parameter but a fundamental determinant of brain function, psychiatric symptom severity, and response to psychological interventions (1, 2). Within this framework, anorexia nervosa (AN) represents a paradigmatic condition in which severe and prolonged malnutrition creates a neurobiological substrate that actively perpetuates psychiatric dysfunction: energy deprivation depletes neurotransmitter precursors, promotes neuroinflammation, disrupts the gut-brain axis, and alters neuroendocrine signaling in ways that compound the cognitive and affective rigidity characteristic of the disorder (2, 3). This bidirectional relationship,in which undernutrition worsens psychiatric symptoms and psychiatric symptoms sustain undernutrition,implies that nutritional rehabilitation is not merely a treatment component alongside psychotherapy, but its biological prerequisite: without adequate restoration of nutritional status, the neurobiological conditions necessary for durable psychiatric recovery may remain unachievable (4). AN is increasingly recognised as a severe mental illness (SMI) requiring structured psychiatric rehabilitation: among eating disorders, it carries one of the highest psychiatric mortality rates and is associated with profound psychosocial disability, including severely impaired functioning, social withdrawal, and exercise-related behavioral dysregulation that collectively define a chronic and disabling condition warranting the full resources of residential rehabilitation services.

Despite this theoretical consensus, clinical monitoring of nutritional rehabilitation in AN has relied almost exclusively on body weight and body mass index (BMI). While clinically indispensable, these parameters capture only the aggregate outcome of the nutritional rehabilitation process and do not differentiate between the recovery of metabolically distinct body compartments. In particular, BMI does not discriminate between restoration of fat-free mass (FFM) and skeletal muscle mass (SMM),the metabolically active tissues most depleted during AN,and increases in fat mass or body water. This distinction is clinically meaningful: FFM and SMM are proxies for protein nutritional adequacy and anabolic recovery, and their restoration is associated with improved metabolic rate, functional capacity, and reduced relapse risk (5–7). Multi-frequency bioelectrical impedance analysis (BIA) offers a non-invasive, clinically applicable method for tracking changes in body compartment composition during nutritional rehabilitation. Previous studies have demonstrated that BIA-derived parameters, including FFM, SMM, and total body water, change dynamically during refeeding in AN and may capture dimensions of nutritional recovery not reflected by weight alone (8–10). In the context of residential treatment, where monitoring somatic recovery alongside psychological outcomes is both clinically necessary and methodologically feasible, the systematic use of multi-frequency BIA represents an extension of nutritional assessment fully aligned with the aims of nutritional psychiatry.

The prevailing clinical model assumes that restoration of nutritional status is a prerequisite for meaningful psychological improvement, given that malnutrition itself contributes to cognitive rigidity, affective dysregulation, and impaired treatment responsiveness (2). However, whether psychiatric recovery during treatment actually tracks the degree of nutritional restoration remains insufficiently examined. Previous studies of inpatient and residential AN treatment have documented substantial improvements in eating disorder psychopathology, general distress, and functional impairment over relatively short treatment windows (4), but few have directly tested whether the magnitude of these gains correlates with the degree of somatic recovery as measured by body composition parameters. It is therefore possible that nutritional and psychiatric recovery, while interrelated in the longer term, may follow partially dissociable trajectories over the short time frame of structured residential treatment, with psychological change driven partly by environmental and therapeutic factors that operate independently of ongoing somatic restoration.

Compulsive exercise in AN functions both as a behavioral symptom and a metabolic obstacle: by increasing energy expenditure and counteracting caloric restoration, it delays positive energy balance and prolongs the nutritional deficit underlying psychiatric chronicity (11). Reducing exercise dependence during residential treatment may therefore function as a nutritional intervention in its own right, removing a critical behavioral obstacle to anabolic recovery (12, 13). To date, no study has simultaneously examined BIA-derived body composition changes alongside comprehensive measures of eating disorder psychopathology, general psychological distress, functional impairment, and exercise dependence within the same longitudinal clinical framework, nor directly investigated whether the degree of nutritional recovery is associated with the magnitude of psychiatric improvement during residential treatment. Accordingly, the present study was designed both to describe longitudinal changes in body composition and psychopathology during residential AN treatment and to test whether the magnitude of psychiatric improvement is associated with the magnitude of nutritional recovery, specifically, whether changes in BMI, FFM, and SMM track changes in eating disorder psychopathology, global psychological distress, functional impairment, and exercise dependence. This approach addresses a clinically relevant question in nutritional psychiatry: whether somatic and psychiatric recovery in AN are tightly coupled over the short term or may unfold along partially dissociable trajectories. We expected nutritional rehabilitation to co-occur with psychiatric improvement, while also considering the possibility that these domains might not be strictly aligned over a relatively brief residential treatment interval.

## 2 Methods

### 2.1 Participants and setting

This longitudinal observational study was conducted at the Regional Residential Centre for Eating Disorders “Mariconda”, ASL Salerno, Italy. Participants were patients consecutively admitted to the program with a primary diagnosis of AN or atypical AN according to DSM-5 criteria (3). Inclusion criteria were: (1) primary diagnosis of AN or atypical AN; (2) age ≥ 14 years; (3) completion of both T0 and T1 assessments. Patients were excluded if they presented with binge eating disorder, obesity (BMI ≥ 30 kg/m²), or severe medical comorbidities precluding BIA assessment. The final sample consisted of 19 patients, all female, with a mean age of 20.4 ± 5.6 years (range 14–40) and mean illness duration of 17.6 ± 10.3 months (range 6–48). Ten patients (52.6%) presented with the restricting subtype (AN-R; mean BMI 16.1 ± 1.4 kg/m²) and nine (47.4%) with the binge-eating/purging subtype (AN-BP), the majority of whom met criteria for atypical AN with preserved body weight (mean BMI 19.0 ± 2.3 kg/m²). Demographic and clinical characteristics are summarized in **Table 1**.

**Table 1:** Demographic and clinical characteristics at admission (T0; *N*=19).

Characteristic	*N* = 19
Demographics	
Age, years, M ± SD (range)	20.4 ± 5.6 (14–40)
Illness duration, months, M ± SD (range)	17.6 ± 10.3 (6–48)
BMI at admission, kg/m², M ± SD	18.42 ± 3.08
AN subtype, *n* (%)	
AN-Restricting	10 (52.6)
AN-Binge-Purging / Atypical AN	9 (47.4)
Psychiatric comorbidities, *n* (%)	
Major Depressive Disorder	10 (52.6)
Anxiety Disorder	4 (21.1)
Obsessive-Compulsive Disorder	3 (15.8)
Bipolar Disorder	1 (5.3)
Psychosis	1 (5.3)
Pharmacotherapy, *n* (%)	
Vortioxetine 20 mg/day	8 (42.1)
Aripiprazole 5–10 mg/day	3 (15.8)
Sertraline 50–100 mg/day	3 (15.8)
Fluvoxamine 150–200 mg/day	3 (15.8)
Lithium sulfate 186 mg/day	1 (5.3)

M, mean; SD, standard deviation; BMI, body mass index; AN, anorexia nervosa.

For the seven adolescent participants (age 14–17 years), BMI z-scores were computed retrospectively using the WHO 2007 BMI-for-age reference for girls (14), and compared against the Italian SIEDP-2006 centile charts (15) as an age-specific supplementary anthropometric index. Results are presented in **Supplementary Table 1**. Z-scores at T0 ranged from −2.49 (age 14, AN-R) to +1.54 (age 17, atypical AN), with a mean of −0.64 ± 1.28, reflecting the same diagnostic heterogeneity observed in the full sample. One patient (age 14, BMI 15.0) fell below the 3rd Italian centile; three patients had z-scores between −2 and −1; two had z-scores above the median, consistent with atypical AN at preserved body weight.

Clinical assessments were conducted at two time points: T0 (admission) and T1 (discharge, following a residential stay of approximately five months). The residential program comprised a multidisciplinary treatment approach consistent with the Italian National Guidelines for Nutritional Rehabilitation in Eating Disorders (16), including supervised nutritional rehabilitation with individualized caloric plans (targeting progressive increases in caloric intake in accordance with guideline recommendations), structured limitation of physical activity, psychiatric care, individual and group psychotherapeutic interventions, and structured daily routines aimed at reducing maladaptive behaviors and promoting recovery.

The residential programme delivered the following structured interventions: (1) Individual psychotherapy: weekly sessions (60 minutes) provided by one of four clinical psychologists of the multidisciplinary team, each assigned to one patient. The theoretical orientation was either Cognitive Behavioural Therapy (CBT) or Systemic-Relational therapy, based on the psychologist’s training and the patient’s clinical presentation. (2) Group CBT-based psychotherapy: weekly group sessions (60 minutes) targeting eating disorder cognitions, body image disturbance, interpersonal skills, and relapse prevention. (3) Psychiatric evaluation and pharmacotherapy: weekly psychiatric review with medication management as clinically indicated. (4) Nutritional counselling: sessions with a clinical dietitian delivering individualised caloric plans consistent with Italian National Guidelines (16). (5) Structured daily activities: supervised meals, body-awareness activities, and social skills training embedded in the daily residential schedule.

Patients with AN-R, AN-BP, and atypical AN were included in a single analytical cohort because all received the same residential treatment protocol and presented with comparable baseline psychopathological severity (see **Supplementary Table 2**). Subtype-stratified analyses were not feasible given the subsample sizes (n = 9–10) and are not reported.

As this was an exploratory longitudinal observational study based on consecutive admissions rather than a pre-planned confirmatory trial, no *a priori* sample size calculation was performed. The final sample (N = 19) represents all patients who met inclusion criteria and completed both T0 and T1 assessments during the study period. The inferential capacity of the sample is addressed by the *post-hoc* power analysis reported in Section 2.4. No participant was excluded after enrolment. Missing data were instrument-specific and limited: the BUT-A was missing for one participant at T1 (N = 18 for BUT analyses), and EDS-R data were missing for two participants at T1 (N = 17 for EDS-R analyses) due to clinical scheduling constraints. No imputation was performed; available-case analysis was applied throughout.

Diagnoses were established by psychiatrists and clinical psychologists of the multidisciplinary team using DSM-5 criteria (3). Eating disorder diagnosis was confirmed through the Eating Disorder Examination (EDE; 17), a semi-structured clinician-administered interview considered the gold standard for diagnostic assessment in eating disorder populations. Psychiatric comorbidities were assessed using the Structured Clinical Interview for DSM Disorders, 5th edition (SCID-5; 18).

All participants presented with at least one comorbid psychiatric diagnosis confirmed via SCID-5. The distribution of psychiatric comorbidities was: Major Depressive Disorder (n = 10, 52.6%), Anxiety Disorder (n = 4, 21.1%), Obsessive-Compulsive Disorder (n = 3, 15.8%), Bipolar Disorder (n = 1, 5.3%), and Psychosis (n = 1, 5.3%). The presence of high rates of affective and anxiety comorbidities is consistent with the well-documented psychiatric complexity of residential AN populations and should be considered when interpreting the broad psychopathological improvements observed across SCL-90 dimensions.

All participants received pharmacological treatment throughout the residential programme, prescribed by the treating psychiatrist based on clinical indication and targeting comorbid psychiatric diagnoses. The pharmacotherapy profile was: vortioxetine 20 mg/day (n = 8, 42.1%), aripiprazole 5–10 mg/day (n = 3, 15.8%), sertraline 50–100 mg/day (n = 3, 15.8%), fluvoxamine 150–200 mg/day (n = 3, 15.8%), and lithium sulfate 186 mg/day (n = 1, 5.3%). Medications were maintained at stable doses throughout the treatment period unless clinically indicated otherwise. Pharmacological heterogeneity is acknowledged as a potential confounding factor in the interpretation of psychiatric outcomes (see Section 4.5). In particular, several psychotropic agents prescribed in this sample, including aripiprazole (n = 3) and lithium sulfate (n = 1),carry a documented pharmacological risk of weight gain, which may have contributed to the modest BMI increases observed and cannot be disentangled from the effects of nutritional rehabilitation in the present dataset. This pharmacological confounding is particularly relevant given the small absolute BMI change (mean ΔBMI = +0.27 kg/m²) and limits the degree to which weight gain can be attributed solely to the nutritional rehabilitation programme. Future studies should prospectively track medication type, dose, and duration as covariates in body composition analyses.

### 2.2 Body composition assessment

Body composition was assessed using the Accuniq BC380 multi-frequency segmental BIA device (Selvas Healthcare, Korea), operating at three frequencies (5, 50, and 250 kHz), with 8 tactile electrodes for total body and segmental analysis. The device is validated against DEXA and holds CE Medical Device certification (Class IIa, Directive 93/42). Parameters extracted were: BMI (kg/m²), FFM (kg), SMM (kg), FM (kg and %), TBW (litres), and basal metabolic rate (BMR, kcal/die; estimated by the Accuniq BC380’s integrated predictive algorithm, based primarily on fat-free mass, age, sex, and height, consistent with Müller-type equations validated for underweight populations). It should be noted that device-derived BMR represents a predicted rather than measured value; future studies should integrate indirect calorimetry (e.g., canopy or ventilated hood systems) to provide criterion-standard resting energy expenditure alongside BIA-derived body composition data in AN rehabilitation monitoring. Measurements were obtained under standardized conditions: morning, after overnight fast, following bladder emptying, and after at least 10 minutes of standing rest, applied identically at both time points.

### 2.3 Psychometric measures

Psychopathology, psychological functioning, and body image were assessed using validated Italian versions of six standardized instruments. Brief descriptions are provided below; full psychometric details are provided in **Supplementary Material** (**Supplementary Methods**). All instruments were administered at T0 and T1 by trained clinicians under standardized conditions.

For each instrument, the established age range for validated use is noted: EDE-Q (≥12 years; 19, 20); EDI-3 (≥13 years; 21); SCL-90-R (≥13 years; 22); CIA (primarily adult, clinical use documented from ≥14 years; 23); EDS-R (≥15 years; 24); BUT (≥14 years; 25). The use of the CIA in participants aged 14–15 years without age-specific norms is acknowledged as a limitation in Section 4.5.

Internal consistency values cited for each instrument reflect those reported in the original validation studies and Italian adaptations, as item-level data were not retained in the clinical database used for this study; sample-level calculation of Cronbach’s α or McDonald’s ω was therefore not feasible. This is a recognised limitation of retrospective clinical observational research using standardised scoring software.

The Eating Disorder Examination Questionnaire (EDE-Q 6.0; 26, 27) is a 28-item self-report questionnaire assessing the frequency and severity of eating disorder behaviors and cognitions over the preceding 28 days. It yields four subscales, Restraint, Eating Concern, Shape Concern, and Weight Concern, each scored on a 7-point Likert scale (0–6), with a Global Score computed as the unweighted mean of all four subscales (higher scores indicate greater severity; score ≥2.77 is the established clinical threshold). The EDE-Q demonstrates robust psychometric properties across clinical and community samples, with internal consistency coefficients (Cronbach’s alpha) ranging from 0.78 to 0.93 across subscales, good convergent validity with the interviewer-administered EDE, and adequate sensitivity to change during treatment (19, 26). A validated Italian version was used (28).

The Eating Disorder Inventory-3 (EDI-3; 21) is a 91-item self-report instrument comprising 12 primary subscales that assess eating disorder-specific and general psychological constructs. The three eating disorder-specific subscales, Drive for Thinness, Bulimia, and Body Dissatisfaction, combine to form the ED Risk Composite. The nine general psychological subscales, Low Self-Esteem, Personal Alienation, Interpersonal Insecurity, Interpersonal Alienation, Interoceptive Deficits, Emotional Dysregulation, Perfectionism, Asceticism, and Maturity Fears, are organized into four composite scores: Ineffectiveness (Low Self-Esteem + Personal Alienation), Interpersonal Problems (Interpersonal Insecurity + Interpersonal Alienation), Affective Problems (Interoceptive Deficits + Emotional Dysregulation + Asceticism), and Overcontrol (Perfectionism + Asceticism + Maturity Fears); a General Psychological Maladjustment composite encompasses all nine general subscales. Items are scored on a 6-point Likert scale; results are expressed as age- and sex-referenced T-scores (higher = greater pathology). The EDI-3 demonstrates good internal consistency (Cronbach’s alpha 0.75–0.93 across subscales) and well-established criterion validity in AN populations (21).

The Symptom Checklist-90 (SCL-90-R; 29, 30) is a 90-item self-report instrument assessing a broad range of psychological symptoms on a 5-point Likert scale (0=not at all, 4=extremely). It yields nine primary symptom dimensions, Somatization, Obsessive-Compulsive, Interpersonal Sensitivity, Depression, Anxiety, Hostility, Phobic Anxiety, Paranoid Ideation, and Psychoticism, and three global indices. The Global Severity Index (GSI), used as the primary outcome in the present study, represents the overall level of psychological distress and is the most sensitive single indicator of symptom burden. All scores are expressed as age- and sex-normed T-scores (M = 50, SD = 10); T-scores ≥63 are considered clinically significant. The SCL-90-R has demonstrated good internal consistency (Cronbach’s alpha 0.77–0.90), test-retest reliability, and sensitivity to treatment-related change in eating disorder populations (30).

The Clinical Impairment Assessment (CIA 3.0; 23, 31) is a 16-item self-report questionnaire measuring the degree of psychosocial impairment resulting from eating disorder features over the preceding 28 days. Items are rated on a 4-point Likert scale (0=not at all, 3=a lot), yielding a total score ranging from 0 to 48; a score ≥16 is the established threshold for clinically significant functional impairment in eating disorder samples. The CIA yields three subscale scores, Personal Functioning, Social Functioning, and Cognitive Functioning, though the total score is the primary index. It demonstrates good internal consistency (Cronbach’s alpha 0.94), adequate convergent validity with clinician-rated impairment measures, and sensitivity to treatment-related change (23).

The Exercise Dependence Scale-Revised (EDS-R; 32, 33) is a 21-item self-report instrument assessing the seven DSM-IV criteria for substance dependence as applied to exercise behavior: Tolerance, Withdrawal, Intention Effects, Lack of Control, Time, Reduction of Other Activities, and Continuance. Each subscale comprises three items rated on a 6-point Likert scale (1=never, 6=always), with mean subscale scores computed (range 1–6; higher = greater exercise dependence). Three classification levels are operationally defined: exercise dependence (all subscales ≥5), non-dependence-symptomatic (not all subscales ≥5 but not all ≤3), and non-dependence-asymptomatic (all subscales ≤3). The EDS-R demonstrates good internal consistency (Cronbach’s alpha 0.78–0.92), criterion validity against clinical diagnosis of exercise dependence, and adequate sensitivity to change. The Italian validation (24) confirmed the original seven-factor structure and acceptable psychometric properties in Italian samples.

Descriptive statistics are reported as M ± SD. Four outcomes were pre-specified as primary: EDE-Q Global Score, SCL-90 Global Severity Index (GSI), CIA Total, and EDS-R Total. All other outcomes were designated exploratory secondary. Changes between T0 and T1 were evaluated using the Wilcoxon signed-rank test; effect sizes were calculated as r = Z/√N. Associations between changes in body composition and changes in psychological variables were assessed using Spearman rank correlation coefficients (rs) with 95% confidence intervals. Benjamini-Hochberg false discovery rate (FDR) correction was applied to all secondary outcomes (k = 39). Statistical significance was set at α = 0.05. All analyses were performed using R v4.4.1 (34). A *post-hoc* power analysis indicated that with N = 18 and two-tailed alpha = 0.05, the study had adequate power (≥80%) to detect Spearman correlations of rs ≥ 0.62 or larger; power to detect a medium-sized association (rs = 0.40) was approximately 37%. A sample of approximately N = 47 would be required to achieve 80% power for rs = 0.40. These estimates highlight the exploratory nature of all correlation analyses and the study’s limited capacity to detect small-to-medium associations between nutritional and psychiatric change scores.

The choice of non-parametric statistical methods (Wilcoxon signed-rank test; Spearman’s ρ) was motivated by the small sample size (N = 19) and the results of Shapiro-Wilk normality tests applied to all primary outcome variables and their T0–T1 delta scores. Non-normality was confirmed for several variables at T0 (SCL-90 GSI: W = 0.785, p = 0.001; EDS-R Total: W = 0.893, p = 0.043) and for key delta scores (ΔEDS-R: W = 0.872, p = 0.020; ΔBUT GSI: W = 0.266, p < 0.001), justifying the consistent application of non-parametric procedures across all outcomes.

## 3 Results

### 3.1 Body composition

BMI increased significantly from T0 to T1 (W = 39.0, p = 0.042, r = 0.35; **Table 2a**). The remaining BIA parameters all showed consistent upward trends without reaching statistical significance. FFM increased by +0.39 kg (W = 40.5, p = 0.088, r = 0.31) and SMM by +0.22 kg (W = 34.5, p = 0.083, r = 0.32), both reflecting a trend toward lean mass restoration with small-to-medium effect sizes. FM increased by +0.30 kg (W = 46.0, p = 0.085, r = 0.40), with a medium effect size comparable to BMI, while FM% showed the smallest and least consistent change (W = 67.5, p = 0.268, r = 0.25), indicating that lean and fat compartments increased proportionally. TBW increased by +0.28 L (W = 40.0, p = 0.083, r = 0.40), consistent with the rehydration component of nutritional rehabilitation. Strong inter-BIA correlations confirmed that the modest BMI gain co-occurred with lean mass recovery: ΔBMI, ΔFFM (rs = 0.73, 95% CI [0.40, 0.89], p < 0.001) and ΔBMI, ΔSMM (rs = 0.70, 95% CI [0.35, 0.88], p = 0.001; **Table 3**).

**Table 2a:** BIA body composition parameters at T0 and T1 (Wilcoxon signed-rank test; N = 19).

Parameter	T0 M ± SD	T1 M ± SD	Δ	W	p	r (pFDR)
**BMI (kg/m²)**	18.42 ± 3.08	18.69 ± 3.07	+0.27	39.0	**0.042**	0.35 (0.095)
FFM (kg)	36.70 ± 5.63	37.09 ± 5.30	+0.39	40.5	0.088	0.31 (0.095)
SMM (kg)	20.39 ± 3.04	20.61 ± 2.87	+0.22	34.5	0.083	0.32 (0.095)
FM (kg)	10.25 ± 5.32	10.55 ± 5.39	+0.30	46.0	0.085	0.40 (0.095)
FM (%)	20.67 ± 5.85	21.05 ± 5.57	+0.38	67.5	0.268	0.25 (0.246)
TBW (L)	26.43 ± 4.05	26.71 ± 3.83	+0.28	40.0	0.083	0.40 (0.095)
BMR (kcal/die)	1162.2 ± 124.8	1170.8 ± 117.7	+8.6	40.0	0.080	0.40 (0.095)

W, Wilcoxon statistic; p, raw p-value; r, effect size (Z/√N); pFDR, Benjamini-Hochberg corrected p (k = 39 secondary tests); Bold, p < 0.05. BMI, body mass index; FFM, fat-free mass; SMM, skeletal muscle mass; FM, fat mass; TBW, total body water; BMR, basal metabolic rate (algorithmically derived from BIA parameters by the device software; rs with ΔFFM = 1.00).

**Table 2b:** Pre-specified primary psychiatric outcomes at T0 and T1 (N = 19).

Primary outcome*	T0 M ± SD	T1 M ± SD	Δ	W	p	r
EDE-Q Global Score	3.81 ± 1.16	1.98 ± 1.14	–1.83	0.0	**< 0.001**	**0.74**
SCL-90 GSI (T-score)	69.17 ± 6.31	54.17 ± 8.00	–15.0	5.0	**0.001**	**0.66**
CIA Total	32.33 ± 9.41	19.44 ± 7.48	–12.9	6.0	**0.001**	**0.62**
EDS-R Total (1–6 scale)	3.39 ± 1.30	1.67 ± 1.05	–1.72	0.0	**0.001**	**0.72**

All primary outcomes were pre-specified; no multiple comparison correction applied. EDE-Q, Eating Disorder Examination Questionnaire; SCL-90 GSI, Symptom Checklist-90 Global Severity Index; CIA, Clinical Impairment Assessment; EDS-R, Exercise Dependence Scale-Revised. Bold values indicate p < 0.05. No multiple comparison correction was applied to pre-specified primary outcomes.

**Table 3:** Spearman rank correlations (rs) between change scores in nutritional and psychiatric outcomes (N = 19).

Variable pair	rs	95% CI	p
**ΔBMI, ΔFFM**	**0.73**	[0.40, 0.89]	**< 0.001**
**ΔBMI, ΔSMM**	**0.70**	[0.35, 0.88]	**0.001**
**ΔEDE-Q, ΔSCL-90 GSI**	**0.53**	[0.08, 0.80]	**0.022**
ΔEDE-Q, ΔCIA	0.24	[−0.25, 0.62]	0.346
ΔEDE-Q, ΔEDS-R	0.36	[−0.13, 0.70]	0.138
ΔBMI, ΔEDE-Q	0.06	[−0.42, 0.51]	0.806
ΔBMI, ΔSCL-90 GSI	−0.12	[−0.56, 0.37]	0.638
ΔBMI, ΔCIA	0.14	[−0.35, 0.57]	0.583
ΔBMI, ΔEDS-R	0.16	[−0.33, 0.58]	0.521
ΔFFM, ΔEDE-Q	−0.01	[−0.47, 0.46]	0.971
ΔSMM, ΔEDE-Q	−0.02	[−0.48, 0.45]	0.935
ΔFFM, ΔSCL-90 GSI	0.14	[−0.35, 0.57]	0.591
ΔFFM, ΔCIA	0.06	[−0.42, 0.51]	0.827
ΔFFM, ΔEDS-R	0.25	[−0.25, 0.64]	0.324
ΔSMM, ΔSCL-90 GSI	0.15	[−0.34, 0.58]	0.540
ΔSMM, ΔCIA	0.10	[−0.38, 0.54]	0.691
ΔSMM, ΔEDS-R	0.27	[−0.22, 0.66]	0.272
ΔFM, ΔEDE-Q	0.06	[−0.42, 0.51]	0.819
ΔFM, ΔSCL-90 GSI	−0.32	[−0.69, 0.17]	0.194
ΔFM, ΔCIA	0.13	[−0.36, 0.56]	0.618
ΔFM, ΔEDS-R	0.03	[−0.45, 0.49]	0.916
ΔFM%, ΔEDE-Q	−0.09	[−0.54, 0.39]	0.717
ΔFM%, ΔSCL-90 GSI	−0.46	[−0.76, 0.01]	0.057
ΔFM%, ΔCIA	0.10	[−0.38, 0.54]	0.686
ΔFM%, ΔEDS-R	−0.09	[−0.54, 0.39]	0.719
ΔTBW, ΔEDE-Q	−0.03	[−0.49, 0.44]	0.912
ΔTBW, ΔSCL-90 GSI	0.12	[−0.37, 0.56]	0.636
ΔTBW, ΔCIA	0.06	[−0.42, 0.51]	0.825
ΔTBW, ΔEDS-R	0.23	[−0.26, 0.63]	0.356

95% CIs computed using Fisher z-transformation. Bold = p < 0.05. Δ denotes T0–T1 change score.

**Figure 1:**
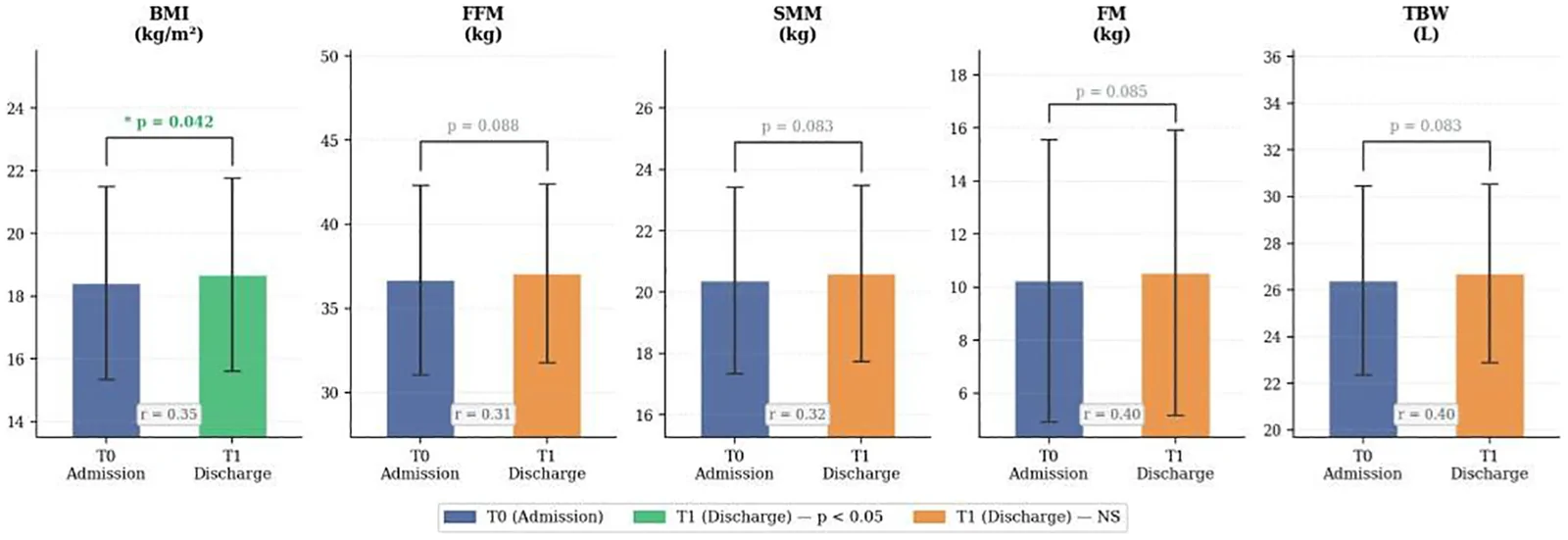
BIA body composition parameters (mean ± SD) at T0 (Admission) and T1 (Discharge). Significance markers indicate Wilcoxon p-values. Effect size r shown in inset badges. Note consistent upward trends across all parameters despite statistical significance reached only for BMI.

**Figure 1** illustrates the directional consistency of changes across all BIA parameters: despite statistical significance being reached only for BMI, all five parameters showed upward trends at T1, underscoring a coherent pattern of nutritional recovery that was modest in absolute magnitude but uniform in direction.

### 3.2 Primary psychiatric outcomes

All four pre-specified primary outcomes improved with large and statistically significant effect sizes (**Table 2b**). EDE-Q Global Score decreased from 3.81 ± 1.16 to 1.98 ± 1.14 (p < 0.001, r = 0.74); the mean T1 score fell below the established clinical threshold of 2.77, consistent with group-level remission of eating disorder psychopathology. SCL-90 GSI decreased from 69.17 ± 6.31 to 54.17 ± 8.00 (p = 0.001, r = 0.66). CIA decreased from 32.33 ± 9.41 to 19.44 ± 7.48 (p = 0.001, r = 0.62). EDS-R Total decreased from 3.39 ± 1.30 to 1.67 ± 1.05 (p = 0.001, r = 0.72); mean T1 scores fell below the published symptomatic threshold of 3.0, indicating a shift toward asymptomatic exercise dependence at the group level.

**Figure 2:**
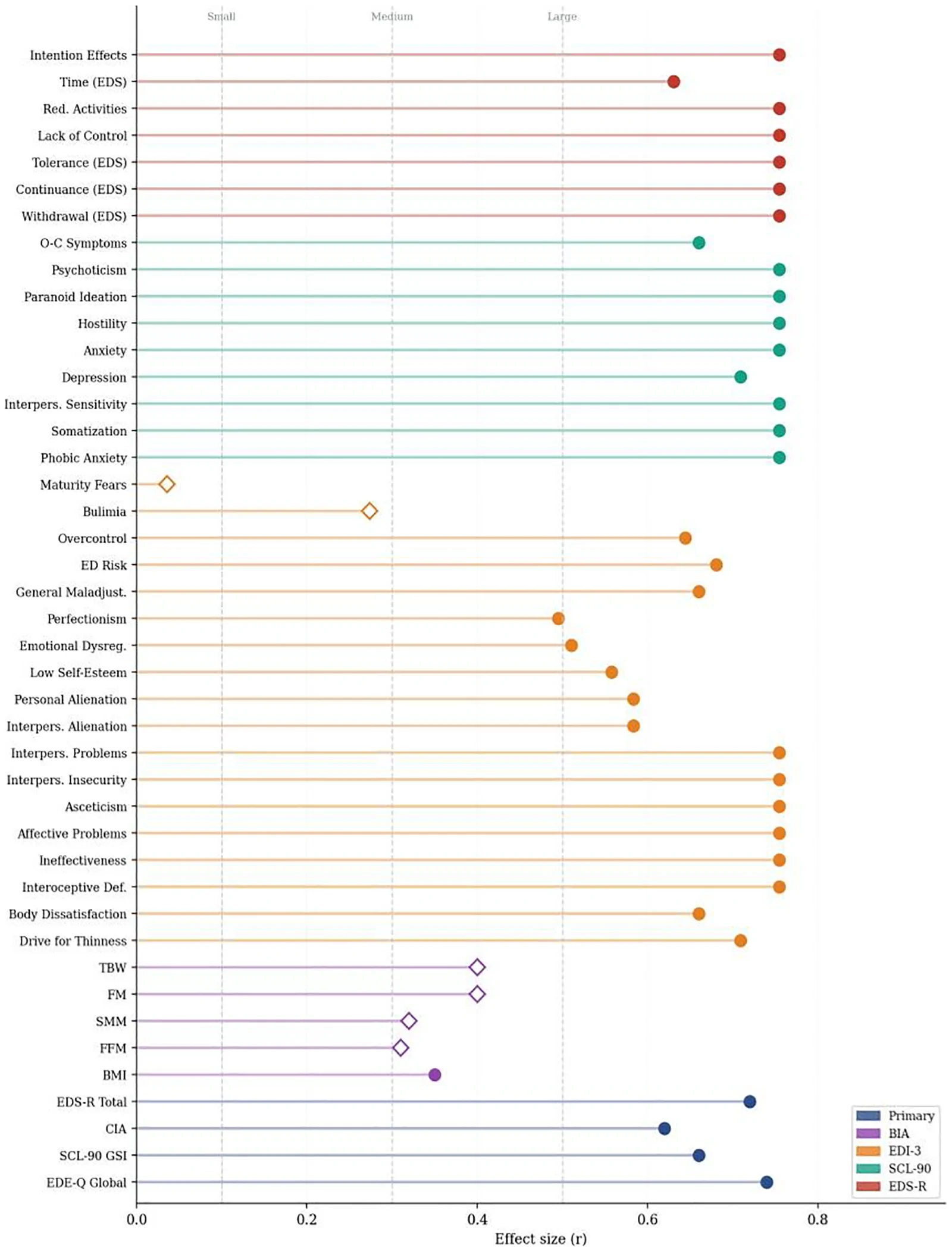
Effect sizes (Wilcoxon r) for all outcome variables, organized by domain. Filled symbols = FDR-corrected p < 0.05. Open circles = raw p < 0.05 only. Diamonds = non-significant. Dashed vertical lines: small (r = 0.10), medium (r = 0.30), large (r = 0.50) thresholds.

**Figure 2** provides a visual summary of Wilcoxon effect sizes (r) across all outcome domains. The figure highlights the marked contrast between the large effect sizes observed in the psychiatric domain, with primary outcomes ranging from r = 0.62 (CIA) to r = 0.74 (EDE-Q), and the majority of EDI-3, SCL-90, and EDS-R subscales in the large range (r ≥ 0.50),and the small-to-medium effect sizes for BIA parameters (r = 0.25–0.40, none FDR-significant). This visual dissociation between somatic and psychiatric effect sizes provides an intuitive representation of the central finding: large and robust psychiatric improvement co-occurred with comparatively modest and statistically non-significant nutritional gains.

### 3.3 Associations between nutritional recovery and psychiatric outcomes

Spearman rank correlations between change scores in nutritional and psychiatric outcome variables are presented in **Table 3**. No significant association was found between any BIA-derived body composition change score and any primary psychiatric outcome. Specifically, ΔBMI was not significantly correlated with ΔEDE-Q Global Score, ΔSCL-90 GSI, ΔCIA Total, or ΔEDS-R Total (all rs values and 95% confidence intervals are reported in **Table 3**; all p > 0.05). Similarly, ΔFFM and ΔSMM were not significantly associated with ΔEDE-Q Global Score. These findings indicate that, within this residential treatment episode, the degree of nutritional recovery,as indexed by change in BMI, fat-free mass, or skeletal muscle mass,was not associated with the magnitude of psychiatric improvement in any primary outcome domain.

Among psychiatric change scores, ΔEDE-Q correlated significantly with ΔSCL-90 GSI (rs = 0.53, 95% CI [0.08, 0.80], p = 0.022), indicating that patients with greater eating disorder psychopathology improvement also showed greater reductions in global psychological distress. ΔEDE-Q did not correlate significantly with ΔEDS-R (rs = 0.36, 95% CI [−0.13, 0.70], p = 0.138) or ΔCIA (rs = 0.24, 95% CI [−0.25, 0.62], p = 0.346).

### 3.4 EDI-3 subscales

Sixteen of 18 EDI-3 subscales showed significant improvement, all surviving FDR correction (**Table 4**). Non-significant subscales were Bulimia (pFDR = 0.246) and Maturity Fears (pFDR = 0.875).

**Table 4:** EDI-3 subscales at T0 and T1 with FDR correction (exploratory secondary; N = 14–19).

EDI-3 Subscale	T0 M ± SD	T1 M ± SD	W	p (raw)	p (FDR)
**Drive for Thinness**	78.2 ± 18.6	58.5 ± 14.6	12.0	0.002	**0.004**
Bulimia	53.0 ± 37.3	41.1 ± 34.5	39.0	0.233	0.246
**Body Dissatisfaction**	80.8 ± 15.8	60.3 ± 21.9	19.0	0.004	**0.006**
**ED Risk Composite**	80.3 ± 11.6	66.1 ± 17.5	1.0	0.003	**0.005**
**Low Self-Esteem**	80.5 ± 16.6	60.8 ± 18.9	25.0	0.015	**0.020**
**Personal Alienation**	86.6 ± 11.9	69.8 ± 20.5	9.0	0.011	**0.015**
**Interpersonal Insecurity**	82.1 ± 15.1	63.4 ± 17.0	0.0	< 0.001	**0.002**
**Interpersonal Alienation**	72.3 ± 24.2	53.6 ± 28.1	9.0	0.011	**0.015**
**Interoceptive Deficits**	89.0 ± 7.5	71.6 ± 16.0	6.0	0.001	**0.002**
**Emotional Dysregulation**	69.8 ± 22.3	49.1 ± 25.8	17.0	0.026	**0.032**
**Perfectionism**	73.4 ± 20.7	65.4 ± 17.4	36.0	0.031	**0.037**
**Asceticism**	82.6 ± 18.1	66.6 ± 22.9	1.0	< 0.001	**0.002**
Maturity Fears	62.6 ± 29.1	57.9 ± 27.2	50.0	0.875	0.875
**Ineffectiveness**	85.7 ± 10.7	63.0 ± 15.0	9.5	0.001	**0.002**
**Interpersonal Problems**	82.3 ± 15.3	62.8 ± 20.4	1.5	0.001	**0.002**
**Affective Problems**	86.2 ± 8.5	63.4 ± 15.3	3.0	< 0.001	**0.002**
**Overcontrol**	83.6 ± 12.8	72.8 ± 17.2	5.0	0.005	**0.008**
**General Maladjustment**	80.3 ± 21.3	61.7 ± 15.9	19.0	0.004	**0.006**

T-scores (higher = greater severity). p (FDR) = Benjamini-Hochberg corrected across k = 39 secondary tests. Bold = FDR-corrected p < 0.05; plain text = not FDR-significant. **Supplementary Table 3** (EDI-3 subscales) is provided in the **Supplementary Material** file.

### 3.5 SCL-90 subscales

All nine SCL-90 dimensions improved significantly, all surviving FDR correction (**Table 5**). Phobic Anxiety was the highest-scoring dimension at T0 (74.1 ± 2.8) and remained highest at T1 (60.5 ± 9.0) despite significant improvement.

**Table 5:** SCL-90 subscales at T0 and T1 with FDR correction (exploratory secondary; N = 18).

SCL-90 Subscale	T0 M ± SD	T1 M ± SD	W	p (raw)	p (FDR)
Somatization (SOM)	68.7 ± 8.0	52.9 ± 7.6	7.5	0.001	**0.002**
Obsessive-Compulsive (O-C)	64.4 ± 9.9	52.1 ± 9.7	15.0	0.004	**0.006**
Interpersonal Sensitivity	65.7 ± 8.8	52.7 ± 8.6	9.5	0.001	**0.002**
Depression (DEP)	67.2 ± 7.8	53.8 ± 8.0	12.5	0.002	**0.003**
Anxiety (ANX)	67.8 ± 7.7	54.7 ± 7.0	3.0	< 0.001	**0.002**
Hostility (HOS)	65.9 ± 7.7	52.6 ± 8.0	3.0	< 0.001	**0.002**
Phobic Anxiety (PHOB)	74.1 ± 2.8	60.5 ± 9.0	0.0	0.001	**0.002**
Paranoid Ideation (PAR)	66.9 ± 7.4	52.8 ± 7.8	5.0	< 0.001	**0.002**
Psychoticism (PSY)	70.8 ± 5.8	55.6 ± 9.2	3.5	0.001	**0.002**
GSI (global)	69.2 ± 6.3	54.2 ± 8.0	5.0	0.001	—

T-scores. GSI (global) was a pre-specified primary outcome (FDR not applicable). All subscales significant at raw and FDR-corrected levels. **Supplementary Table 4** (SCL-90 subscales) is provided in the **Supplementary Material** file. Bold values indicate FDR-corrected p < 0.05.

**Figure 3:**
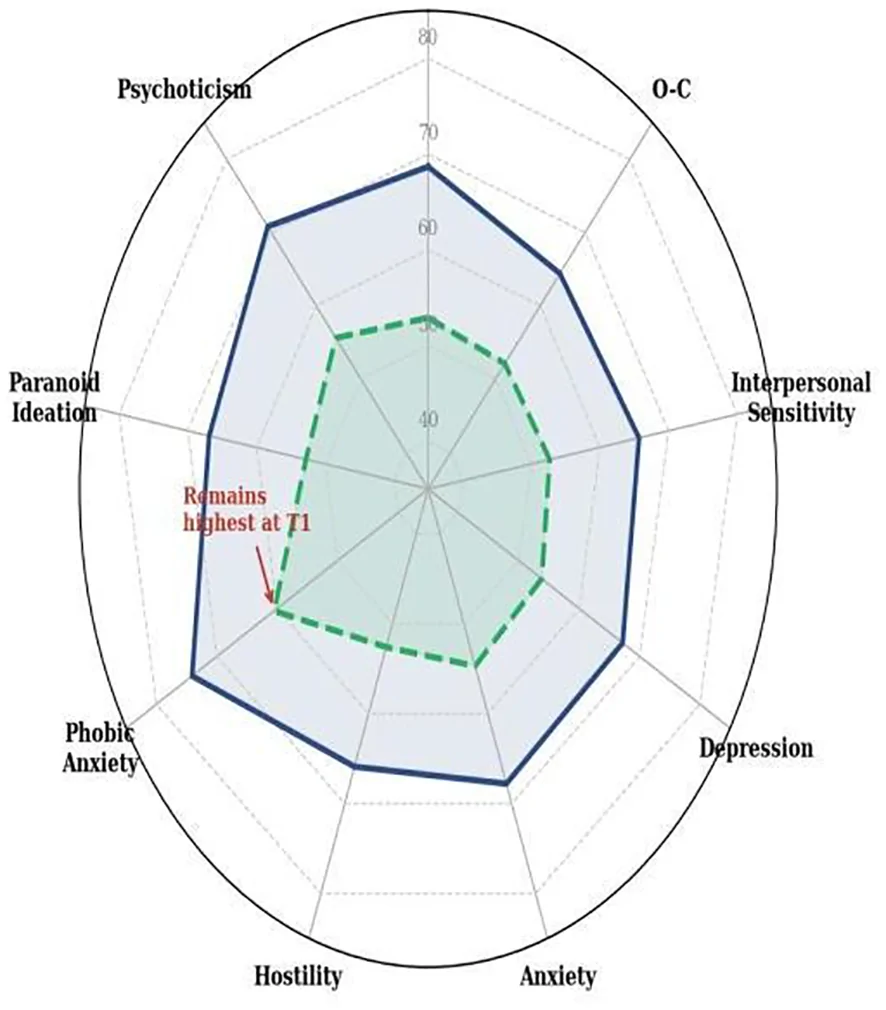
SCL-90 symptom profile (radar chart) at T0 and T1. T-scores (higher = greater severity). All nine dimensions improved significantly (all p ≤ 0.004; all FDR p ≤ 0.006). Phobic Anxiety remained the highest-scoring dimension at T1.

The radar chart in **Figure 3** provides a visual representation of the SCL-90 symptom profile at T0 and T1 across all nine dimensions simultaneously. The figure shows a uniform inward contraction of the profile polygon from admission to discharge, reflecting the global and relatively homogeneous nature of symptom reduction. Notably, the angular prominence corresponding to Phobic Anxiety remains the largest projection at both time points, illustrating its persistence as the residual dominant dimension despite clinically meaningful improvement.

### 3.6 EDS-R subscales

All seven EDS-R subscales showed significant improvement, all surviving FDR correction (**Table 6**). Withdrawal showed the largest reduction; the Time subscale showed the smallest reduction while still significant (p = 0.006, pFDR = 0.007).

**Table 6:** EDS-R subscales at T0 and T1 with FDR correction (exploratory secondary; N = 17).

EDS-R Subscale	T0 M ± SD	T1 M ± SD	W	p (raw)	p (FDR)
Withdrawal	3.67 ± 1.33	1.77 ± 0.90	0.0	< 0.001	**0.002**
Continuance	3.11 ± 1.20	1.56 ± 1.01	0.0	0.001	**0.002**
Tolerance	3.39 ± 1.38	1.67 ± 1.00	0.0	0.001	**0.002**
Lack of Control	3.28 ± 1.56	1.50 ± 0.83	0.0	0.001	**0.002**
Reduction of Activities	3.06 ± 1.22	1.56 ± 1.01	0.0	0.001	**0.002**
Time	3.11 ± 1.52	1.67 ± 1.05	2.0	0.006	**0.007**
Intention Effects	3.28 ± 1.45	1.61 ± 1.01	0.0	0.001	**0.002**

Mean item scores on 1–6 Likert scale. All subscales significant at raw and FDR-corrected levels. **Supplementary Table 5** (EDS-R subscales) is provided in the **Supplementary Material** file. Bold values indicate FDR-corrected p < 0.05.

**Figure 4:**
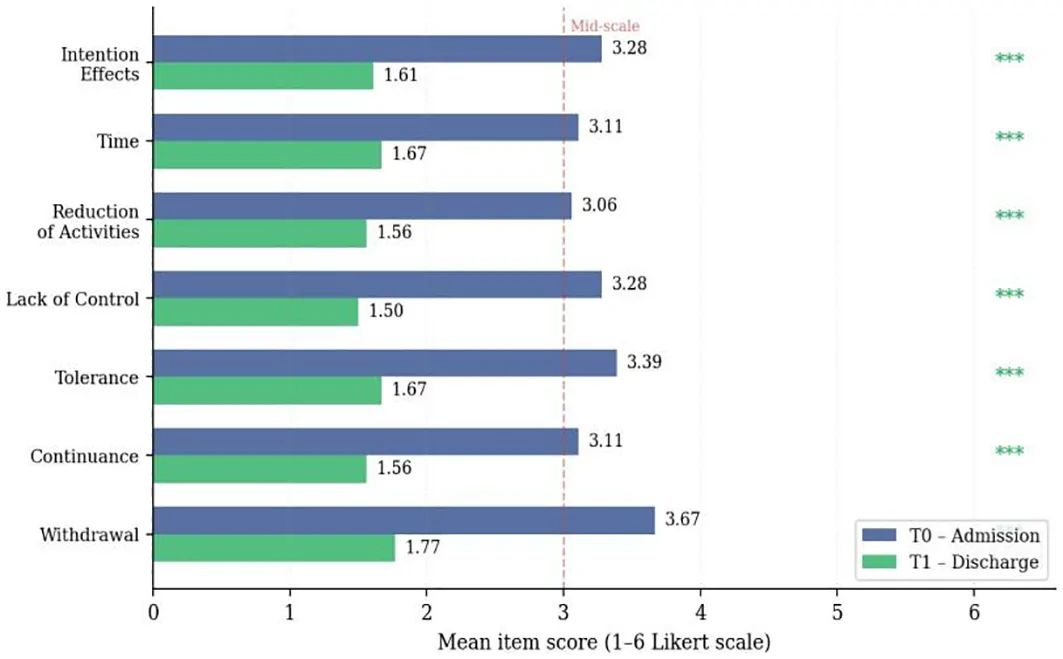
EDS-R subscales: mean item scores at T0 and T1 (N = 17). Dashed line = mid-scale value (3.0). *** = p < 0.01.

**Figure 4** displays mean item scores for each EDS-R subscale at T0 and T1, with a dashed reference line at the mid-scale value (3.0). At T0, all seven subscale means were at or above the mid-scale reference, indicating clinically meaningful exercise dependence tendencies across all DSM-IV-derived criteria. At T1, all subscale means had fallen clearly below the mid-scale reference, representing a substantial and clinically meaningful normalization of perceived exercise dependence. The figure also illustrates the relative uniformity of improvement across subscales, with Withdrawal showing the largest absolute reduction and Time the smallest.

### 3.7 Body image disturbance: BUT-A subscales

All seven BUT-A indices showed large and statistically significant improvements from T0 to T1, all surviving FDR correction (**Table 7**). The GSI showed the largest effect size in the entire study (W = 0.0, p < 0.001, r = 0.88), followed by Weight Phobia and Body Image Concern (both r = 0.81, both p < 0.001). Depersonalization (r = 0.73), Compulsive Self-Monitoring (r = 0.71), and PSDI (r = 0.78) also showed large-magnitude improvements. Avoidance showed the smallest effect size, though still large and FDR-significant (r = 0.65, p = 0.006). ΔBMI was not significantly associated with ΔBUT GSI (rs = 0.23, 95% CI [−0.26, 0.63], p = 0.352), consistent with the pattern of somatic-psychiatric dissociation observed across all primary outcomes.

**Table 7:** BUT-A subscales at T0 and T1 with FDR correction (exploratory secondary; N = 18).

BUT-A Subscale	T0 M ± SD	T1 M ± SD	W	p (raw)	p (FDR)	r
Weight Phobia	4.06 ± 0.57	2.93 ± 1.06	2.0	< 0.001	< 0.001	0.81
Body Image Concern	3.61 ± 0.73	2.49 ± 1.04	7.0	< 0.001	< 0.001	0.81
Avoidance	2.54 ± 1.04	1.59 ± 1.06	22.0	0.006	0.006	0.65
Comp. Self-Monitoring	2.94 ± 1.08	1.88 ± 1.06	16.0	0.003	0.003	0.71
Depersonalization	3.01 ± 0.80	1.82 ± 1.12	14.0	0.002	0.003	0.73
GSI	3.33 ± 0.55	2.02 ± 0.86	0.0	< 0.001	< 0.001	0.88
PSDI	3.41 ± 0.88	2.46 ± 1.00	10.0	0.001	0.002	0.78

Items rated 0–5 (higher = greater body image disturbance). p (FDR) = Benjamini–Hochberg corrected across k = 7 BUT tests. GSI, Global Severity Index; PSDI, Positive Symptom Distress Index. All subscales FDR-significant. N = 18. **Supplementary Table 6** (BUT-A subscales) is provided in the **Supplementary Material** file.

## 4 Discussion

The present study examined longitudinal changes in body composition and psychopathological functioning during five months of residential treatment for anorexia nervosa and atypical AN. The central finding is a dissociation between the two domains: improvements in eating disorder psychopathology, general psychological distress, functional impairment, exercise dependence, and body image disturbance were consistently large in magnitude, whereas changes in BIA-derived body composition parameters were modest and not statistically associated with psychiatric outcomes. Critically, given that the study had only 37.5% power to detect a medium-sized association (rs = 0.40) and 80% power only for large associations (rs ≥ 0.62), the absence of significant correlations cannot be interpreted as evidence that nutritional and psychiatric recovery are genuinely dissociated. Rather, the present findings are best understood as hypothesis-generating: they are consistent with the possibility of partial independence between somatic and psychological trajectories over this treatment window, but they are equally consistent with a Type II error in a substantially underpowered sample. These findings should be interpreted as preliminary and hypothesis-generating given the small sample size, diagnostic heterogeneity, and single-site observational design.

### 4.1 Nutritional recovery: what BIA adds beyond BMI

BMI increased modestly but significantly over the treatment period (ΔBMI = +0.27 kg/m², p = 0.042, r = 0.35), while changes in fat-free mass and skeletal muscle mass showed consistent directional trends but did not reach statistical significance, partly reflecting the limited statistical power available with N = 19. These findings are consistent with the literature on residential treatment for AN, in which meaningful body composition restoration typically requires extended rehabilitation periods beyond five months (5, 7). The multi-frequency BIA approach nonetheless adds informative detail beyond BMI alone: the strong correlations between ΔBMI and ΔFFM (rs = 0.73) and ΔSMM (rs = 0.70) confirm that the modest weight gain was predominantly lean mass recovery rather than fat deposition,a clinically meaningful distinction that BMI alone cannot capture (8, 9).

The fat compartment and total body water warrant individual comment. Fat mass increased by+0.30 kg with a medium effect size (r = 0.40), indicating a genuine trend toward adipose tissue restoration despite failing to reach significance. The preservation of comparable effect sizes across FM, TBW, FFM, and SMM (all r = 0.31–0.40) confirms uniform, compartment-proportional recovery rather than selective lean or fat accretion,a pattern consistent with early-phase refeeding physiology. FM% showed the weakest trend (r = 0.25), reflecting that lean and fat mass increased proportionally, leaving the relative composition largely unchanged. The most noteworthy single correlation in the full BIA × psychiatric matrix was ΔFM%, ΔSCL-90 GSI (rs = −0.46, 95% CI [−0.76, 0.01], p = 0.057): patients with proportionally greater body fat gain showed a directional tendency toward greater reduction in global psychological distress. Though not statistically significant and requiring replication, the direction is clinically plausible: adipose tissue restoration in AN is associated with normalization of leptin and reproductive hormones that modulate mood and stress reactivity. TBW increased by +0.28 L (r = 0.40), consistent with the rehydration component of nutritional rehabilitation. In the early months of residential AN rehabilitation, a proportion of apparent FFM gain is attributable to fluid recomposition: as chronically dehydrated tissues are progressively re-perfused, both intracellular and extracellular water compartments expand, transiently inflating BIA-derived FFM estimates before true myofibrillar protein accretion has occurred. This refeeding oedema phenomenon is well-documented in residential AN settings and implies that the FFM trend reported here (ΔFFM +0.39 kg, r = 0.31) likely represents a mixture of genuine lean mass recovery and early rehydration,a distinction that only phase angle or ECW/ICW monitoring can resolve (see Section 4.5).

Changes in BIA parameters were not significantly correlated with changes in any psychiatric outcome,a finding that was quantitatively consistent across all six tested pairings (all rs between −0.12 and 0.16, all p > 0.35). This pattern is consistent with the possibility that, over a relatively short residential treatment episode, somatic and psychological recovery may proceed through at least partially independent pathways. One interpretation is that the structured residential environment,including behavioral containment, regularized eating, activity restriction, and multidisciplinary care,may support psychiatric improvement through mechanisms that operate on a different time scale from biological body composition restoration. However, this interpretation is speculative: the present data do not include measures of treatment process variables, and the observational design does not permit causal inference. The finding could equally reflect insufficient statistical power, a true independence over this particular time window, or unmeasured confounders. Longitudinal designs with multiple assessment points, larger samples, and mediation analyses would be needed to examine these possibilities.

This null finding is interpretable in at least three ways: (1) somatic and psychiatric recovery may genuinely proceed through partially independent pathways over this treatment window; (2) the study was insufficiently powered to detect true associations of small-to-medium magnitude; or (3) the restricted range of nutritional variability,reflecting modest weight gain and diagnostic heterogeneity,may have limited the detectable correlation regardless of the true underlying relationship. The present data cannot distinguish between these possibilities, and the null correlation finding should not be interpreted as confirmatory evidence of independence between nutritional and psychiatric recovery.

The modest nutritional change observed may also reflect a floor effect in nutritional variability: the restricted range of BMI gain (mean ΔBMI = +0.27 kg/m²), partly attributable to the substantial proportion of patients with atypical AN at near-normal body weight, may have attenuated detectable somatic-psychiatric correlations independently of any true biological independence between these domains. Future studies should ensure adequate nutritional recovery before drawing conclusions about the somatic-psychiatric relationship.

The modest observed weight gain warrants contextualisation within the relevant clinical guidelines. BIA-derived basal metabolic rate at admission averaged 1,162 ± 125 kcal/die (25.4 ± 2.9 kcal/kg/die), consistent with the metabolic suppression characteristic of AN and below the guideline-recommended starting caloric intake of 30–40 kcal/kg/die for nutritional rehabilitation (16). ΔBMR was non-significant (+8.6 ± 20.0 kcal/die, W = 40, p = 0.080, r = 0.40), reflecting the minimal degree of metabolic recovery,consistent with and derivable from the non-significant change in fat-free mass (with which BMR is algorithmically colinear in the device output). The Italian National Guidelines for Nutritional Rehabilitation in Eating Disorders (16) recommend a weight recovery rate of 1.0–1.5 kg per week in residential and inpatient settings, achieved through progressive increases in caloric intake (starting from approximately 1,000–1,600 kcal/day and increasing to 70–100 kcal/kg/day during the weight gain phase). The observed mean ΔBMI of +0.27 kg/m² over approximately five months corresponds to an estimated weight gain of roughly 0.03–0.05 kg per week for an average-height female patient,substantially below the guideline target. This gap reflects, in part, the diagnostic composition of the sample: patients with atypical AN at near-normal body weight (n = 9, mean BMI T0 = 19.0 ± 2.3 kg/m²) have limited biological and clinical margin for BMI gain, whereas the AN-R subgroup (n = 10, mean BMI T0 = 16.1 ± 1.4 kg/m²) showed a somewhat larger, though still modest, nutritional gain (ΔBMI +0.41 ± 0.38 vs +0.12 ± 0.63 in atypical AN; Mann-Whitney U = 55, p = 0.435). Importantly, psychiatric improvement was comparable across subtypes (ΔEDE-Q: AN-R −1.66 ± 1.19 vs atypical AN −1.99 ± 1.29, p = 0.596; ΔSCL-90 GSI: −16.1 ± 12.0 vs −13.9 ± 11.0, p = 0.825), suggesting that the observed somatic-psychiatric dissociation was not driven solely by the inclusion of near-normal-weight patients.

### 4.2 Psychiatric recovery in the context of nutritional rehabilitation

Effect sizes for primary psychiatric outcomes (r = 0.62–0.74) were in the large range and compare favorably with those reported in systematic reviews of residential AN treatment (4). Sixteen of 18 EDI-3 subscales, all nine SCL-90 symptom dimensions, and all seven EDS-R subscales survived FDR correction, indicating that improvement was not confined to isolated outcome domains but extended broadly across eating disorder-specific, psychological, and behavioral measures.

The inclusion of the Body Uneasiness Test (BUT-A) provides additional detail on the nature of improvement. All seven BUT-A indices improved significantly and survived FDR correction, with effect sizes ranging from r = 0.65 (Avoidance) to r = 0.88 (GSI). The GSI finding is the largest in the study, though its interpretation warrants some caution given the small sample size and a single data entry error that was identified and corrected prior to analysis. Of clinical interest is the Depersonalization subscale (r = 0.73), which captures feelings of detachment and estrangement from one’s own body,a dimension that intersects with the interoceptive deficits characteristic of AN and may be relevant to treatment-related changes in body awareness (35). These findings extend the characterization of treatment-related improvement to include multidimensional body image disturbance, a core maintaining factor in AN that has received comparatively little systematic measurement in longitudinal residential treatment studies.

*Post-hoc* descriptive subgroup analyses comparing AN-R (n = 10) and atypical AN (n = 9) revealed, as expected, significant differences in baseline somatic parameters (BMI: 16.1 ± 1.5 vs 21.0 ± 2.4, p < 0.001; FFM: 33.4 ± 3.9 vs 40.4 ± 5.4, p = 0.009). However, baseline psychopathological profiles and all psychiatric delta scores were statistically indistinguishable between subtypes (all p > 0.40; see **Supplementary Table 2**), indicating that the observed psychiatric improvements were not attributable to diagnostic subtype composition. Formal subgroup analyses of somatic-psychiatric associations within each diagnostic group were not feasible given the subsample sizes (n = 9–10) and are not reported. The diagnostic heterogeneity of the sample remains an important limitation affecting generalisability of the somatic findings.

The magnitude of psychiatric improvement observed,large effect sizes across all primary and most secondary outcomes within a five-month window,is consistent with the therapeutic architecture of the residential setting itself. Unlike outpatient or semi-residential contexts, full residential treatment provides daily cognitive restructuring, environmental containment of anxiety-provoking stimuli, structured exposure to feared foods, peer group modelling, and continuous limitation of compulsive exercise,all operating simultaneously and continuously across the entire waking day. The particularly large reduction in exercise dependence (EDS-R total: r = 0.72, all seven subscales FDR-significant) is especially instructive in this regard: compulsive exercise in AN is both a behavioral symptom and a metabolic disruptor that actively counteracts nutritional rehabilitation by diverting caloric surplus away from tissue anabolism. Its rapid containment within the residential milieu may therefore create the psychological conditions for symptomatic improvement, anxiety reduction, improved interoceptive awareness, attenuated fear of weight gain,before the somatic tissues have had sufficient biological time to complete cellular anabolism and neurobiological normalization. This offers a mechanistic narrative for the somatic-psychiatric dissociation: the residential environment acts as a rapid psychiatric lever, while true nutritional recovery and its neurobiological downstream effects unfold over a longer, post-discharge timeframe that the present study was not designed to capture.

The absence of significant correlations between BIA change scores and any psychiatric outcome was consistent across primary and exploratory pairings, including body image (ΔBMI, ΔBUT GSI: rs = 0.23, p = 0.352). This does not imply that nutritional and psychiatric recovery are unrelated in a broader sense; rather, it indicates that within this specific clinical context, the magnitude of psychiatric improvement was not proportional to the magnitude of nutritional change. Across the full 24-pair BIA × psychiatric correlation matrix (**Table 3**), one association approached but did not reach the conventional threshold: ΔFM%, ΔSCL-90 GSI (rs = −0.46, 95% CI [−0.76, 0.01], p = 0.057), suggesting a directional tendency for patients who gained proportionally more body fat to show greater reduction in global psychological distress. This finding remains exploratory and hypothesis-generating. The five-month observation window, the modest degree of nutritional change, and the limited statistical power are all factors that could attenuate detectable associations.

### 4.3 Exercise dependence reduction as a nutritional rehabilitation facilitator

All seven EDS-R subscales showed large and statistically significant improvements (r = 0.72, all FDR-corrected). In the nutritional psychiatry framework, compulsive exercise functions as a behavioral confounder: by increasing energy expenditure and counteracting caloric restoration, it can delay positive energy balance and prolong nutritional deficit (11, 36). From this perspective, reduction in perceived exercise dependence may have facilitated the modest nutritional gains observed. The directional association between ΔEDS-R and ΔEDE-Q (rs = 0.36, 95% CI [−0.13, 0.70]), while non-significant, is consistent with the hypothesis that exercise normalization and eating disorder cognitions co-vary during residential treatment, but the wide confidence interval precludes firmer conclusions. It should be noted that the EDS-R measures subjective perceived exercise dependence and was administered in a setting where physical activity was externally restricted; the observed reductions reflect changes in cognitive and perceived exercise dependence rather than objectively measured behavioral change.

### 4.4 Phobic anxiety as a resistant residual target

Among SCL-90 dimensions, Phobic Anxiety showed the highest baseline T-score (74.1 ± 2.8) and remained the highest-scoring dimension at T1 (60.5 ± 9.0), despite statistically significant improvement, and was the only dimension still clearly in the clinically elevated range at discharge. The convergent finding that BUT Depersonalization also remained relatively elevated despite improving significantly suggests that the experiential and fear-based dimensions of body image and anxiety are more refractory to the components of treatment delivered in this program than other symptom dimensions. Proposed mechanisms include conditioned fear responses, interoceptive dysregulation, and reward-avoidance circuits that may require targeted exposure-based work to modify (35). These observations may support the systematic inclusion of exposure-based protocols targeting food- and body-related avoidance within residential curricula, though the present evidence is observational and does not permit conclusions about which specific treatment components are responsible for the differential response.

### 4.5 Strengths and limitations

Strengths include the integration of multi-frequency BIA with a comprehensive psychometric battery spanning eating disorder-specific cognitions (EDE-Q, EDI-3), general psychological distress (SCL-90), functional impairment (CIA), compulsive exercise (EDS-R), and body image disturbance (BUT-A) within a single longitudinal clinical framework. Additional strengths include the rigorously standardized BIA protocol, the pre-specification of primary outcomes, the application of Benjamini-Hochberg FDR correction to all secondary outcomes, and the reporting of 95% confidence intervals for all correlation coefficients, which make the precision of null findings transparent.

The primary limitation is the small sample size (N = 19), which limits statistical power, may produce upwardly biased effect size estimates, and precludes meaningful subgroup or mediation analyses. The absence of significant somatic-psychiatric correlations should be interpreted with this constraint in mind: a true association of small-to-medium magnitude could plausibly exist but remain undetected at this sample size.

The study had only 37.5% power to detect medium-sized somatic-psychiatric associations (rs = 0.40), meaning a Type II error probability of approximately 62.5% for effects of this magnitude. The absence of significant correlations between nutritional and psychiatric change scores should therefore not be interpreted as evidence of genuine independence between these recovery domains.

The pharmacological heterogeneity of the sample,with all patients receiving psychotropic medications targeting comorbid diagnoses (predominantly antidepressants and antipsychotics),represents an uncontrolled confounding factor that precludes attribution of psychiatric improvement to psychotherapeutic and nutritional interventions alone. Future studies should account for medication type and dose in the statistical analysis.

Further uncontrolled confounders include illness duration (range 6–48 months) and baseline psychopathological severity, which were not formally entered as covariates in the primary analyses due to the insufficient sample size for multivariate modelling. Future adequately powered studies should examine whether baseline severity and chronicity moderate the relationship between nutritional recovery and psychiatric improvement.

The age range of the sample (14–40 years) raises a methodological concern regarding BMI interpretation in the adolescent subgroup. Seven of 19 participants were aged 14–17 years, for whom age- and sex-adjusted BMI z-scores are the developmentally appropriate metric rather than absolute BMI values. WHO 2007 BMI-for-age z-scores were therefore computed retrospectively for these seven participants and are reported in **Supplementary Table 1**. Z-scores ranged from −2.49 to +1.54 (mean −0.64 ± 1.28), confirming the diagnostic heterogeneity of this subgroup: one patient fell below the 3rd Italian centile (severe underweight for age), while two had z-scores above the age-specific median (atypical AN with preserved weight). Main analyses were conducted using absolute BMI for consistency across the full age range; the z-score data are provided as supplementary information for transparency.

The comparatively modest degree of nutritional change (mean ΔBMI = +0.27 kg/m²) and the relatively high mean baseline BMI (18.42 kg/m²),reflecting the substantial proportion of patients with atypical AN at near-normal body weight,may have restricted the range of anthropometric variation available to detect correlations with psychiatric outcomes. These sample characteristics should be considered when interpreting the absence of somatic-psychiatric correlations; the findings may not generalize to more severely undernourished samples.

Further limitations include:the single-arm design with no control group or comparator receiving treatment-as-usual (TAU) or alternative modalities, which precludes causal inference about programme-specific effects; the observational design, which precludes causal attribution of improvements to specific treatment components; the absence of post-discharge follow-up, so the durability of gains is unknown; the lack of DEXA validation for BIA, important given that refeeding-related fluid shifts may affect algorithm accuracy; the use of self-report measures for exercise dependence and body image in a setting with structured activity restriction; and the single-site, exclusively female design, which limits external validity. One BUT GSI data entry error (T1 value incorrectly recorded as 238.00 for one patient) was identified and corrected to the clinically.

The assessment window was limited to the residential episode (T0 to T1 at discharge), with no post-discharge follow-up conducted. The long-term durability of the observed psychiatric and nutritional gains therefore remains unknown, and it cannot be determined whether improvements were maintained, consolidated, or reversed after patients returned to community settings. Future studies should incorporate structured follow-up assessments at 3, 6, and 12 months post-discharge to characterise the longitudinal trajectory of recovery.

consistent value of 2.38 following author verification; this correction does not affect any other variable or analysis in the study.

A further limitation specific to the BIA methodology concerns the validity of derived body composition parameters,particularly fat-free mass and skeletal muscle mass,in the context of AN refeeding. BIA-derived compartments depend on proprietary prediction algorithms calibrated in healthy adult populations and may be affected by the refeeding-related fluid redistribution that is characteristic of early nutritional rehabilitation in AN. The phase angle (PhA) and the extracellular-to-intracellular water ratio (ECW/ICW), which are derived directly from raw BIA impedance data and are less algorithm-dependent, are considered more robust indicators of nutritional status and cellular integrity in this population (9). These parameters were not available in the present dataset and should be included in future studies using BIA for nutritional monitoring in eating disorder samples.

## 5 Conclusions

Five months of structured residential treatment were associated with large-magnitude improvements across psychological, behavioral, and nutritional domains in patients with AN and atypical AN. Large effect sizes for all primary outcomes (EDE-Q, SCL-90 GSI, CIA, EDS-R The absence of significant somatic-psychiatric correlations should be interpreted with caution given the study’s limited statistical power (Type II error probability approximately 62.5% for rs = 0.40), and does not permit inference about the degree of coupling or independence between nutritional and psychiatric recovery in AN. total) were accompanied by modest but directionally consistent BIA-derived nutritional gains, with strong correlations between BMI gain and lean mass recovery confirming the qualitative composition of weight restoration. Somatic and psychiatric recovery appear to proceed along partially independent trajectories, consistent with the nutritional psychiatry hypothesis that partial restoration may be sufficient for acute psychological gains while full neurobiological recovery requires extended rehabilitation. The persistence of elevated Phobic Anxiety at discharge highlights the need for more intensive exposure-based interventions targeting food- and body-related fear. All findings should be considered preliminary. Future studies with larger and more homogeneous samples, controlled designs, DEXA validation, objective physical activity measurement, and post-discharge follow-up are essential to characterize the causal relationships between nutritional rehabilitation quality, exercise normalization, and sustained psychiatric recovery in AN.

## Data Availability

The datasets generated and/or analysed during the current study are not publicly available due to privacy and ethical restrictions regarding sensitive clinical data, but are available from the corresponding author upon reasonable request.
