# Supplementary material for: Psychosocial recovery and nutritional rehabilitation in residential treatment for anorexia nervosa: an exploratory longitudinal multi-frequency bioelectrical impedance analysis study
Source: Front Psychiatry. 2026 Sep 3;17:1883192. doi: 10.3389/fpsyt.2026.1883192 (PMC13582624; doi:10.3389/fpsyt.2026.1883192)
Supplement: Supplementary file 1 [file Supplementaryfile1.docx]

**Supplementary Material**

*Psychosocial Recovery and Nutritional Rehabilitation in Residential Treatment for Anorexia Nervosa: An Exploratory Longitudinal Multi-Frequency Bioelectrical Impedance Analysis Study*

**Supplementary Methods: Full Instrument Descriptions**

***Eating Disorder Examination Questionnaire (EDE-Q 6.0)***

The EDE-Q 6.0 (Fairburn & Beglin, 1994, 2008) is a 28-item self-report questionnaire assessing the frequency and severity of eating disorder behaviors and cognitions over the preceding 28 days. It yields four subscales: Restraint, Eating Concern, Shape Concern, and Weight Concern, each scored on a 7-point Likert scale (0–6), with a Global Score computed as the unweighted mean of all four subscales (higher scores indicate greater severity; score ≥ 2.77 is the established clinical threshold for eating disorder psychopathology). Internal consistency coefficients (Cronbach's α) range from 0.78 to 0.93 across subscales; the instrument demonstrates good convergent validity with the interviewer-administered EDE and adequate sensitivity to change during treatment (Fairburn & Beglin, 1994; Mond et al., 2004). A validated Italian version was used (Calugi et al., 2017).

***Eating Disorder Inventory-3 (EDI-3)***

The EDI-3 (Garner, 2004) is a 91-item self-report instrument comprising 12 primary subscales assessing eating disorder-specific and general psychological constructs. The three eating disorder-specific subscales: Drive for Thinness, Bulimia, and Body Dissatisfaction, form the Eating Disorder Risk Composite. The nine general psychological subscales: Low Self-Esteem, Personal Alienation, Interpersonal Insecurity, Interpersonal Alienation, Interoceptive Deficits, Emotional Dysregulation, Perfectionism, Asceticism, and Maturity Fears, are organized into four composite scores: Ineffectiveness, Interpersonal Problems, Affective Problems, and Overcontrol. Items are scored on a 6-point Likert scale; results are expressed as age- and sex-referenced T-scores (M = 50, SD = 10; higher = greater pathology). Internal consistency: Cronbach's α 0.75–0.93 across subscales. Validated Italian version used. Age range: ≥ 13 years.

***Symptom Checklist-90-Revised (SCL-90-R)***

The SCL-90-R (Derogatis, 1977, 1994) is a 90-item self-report instrument assessing psychological symptoms on a 5-point Likert scale (0–4). It yields nine primary symptom dimensions — Somatization, Obsessive-Compulsive, Interpersonal Sensitivity, Depression, Anxiety, Hostility, Phobic Anxiety, Paranoid Ideation, and Psychoticism and three global indices. The Global Severity Index (GSI), used as primary outcome in this study, is the most sensitive single indicator of overall symptom burden. All scores are expressed as age- and sex-normed T-scores (M = 50, SD = 10); T-scores ≥ 63 indicate clinically significant symptom levels. Internal consistency: Cronbach's α 0.77–0.90. Italian normative data from Prunas et al. (2012). Age range: ≥ 13 years.

***Clinical Impairment Assessment (CIA 3.0)***

The CIA 3.0 (Bohn et al., 2008; Bohn & Fairburn, 2008) is a 16-item self-report questionnaire measuring psychosocial impairment resulting from eating disorder features over the preceding 28 days. Items are rated on a 4-point Likert scale (0–3), yielding a total score of 0–48; scores ≥ 16 indicate clinically significant functional impairment. Three subscales assess Personal, Social, and Cognitive Functioning. Internal consistency: Cronbach's α = 0.94; adequate convergent validity and sensitivity to treatment-related change (Bohn et al., 2008). Primarily validated in adult samples; clinical use documented from age ≥ 14 years. Its use in participants aged 14–15 years without age-specific norms is acknowledged as a methodological limitation.

***Exercise Dependence Scale-Revised (EDS-R)***

The EDS-R (Hausenblas & Symons Downs, 2002; Symons Downs et al., 2004) is a 21-item self-report instrument assessing exercise dependence across seven subscales corresponding to DSM-IV substance dependence criteria: Tolerance, Withdrawal, Intention Effects, Lack of Control, Time, Reduction of Other Activities, and Continuance. Each subscale comprises three items rated on a 6-point Likert scale (1 = never, 6 = always); mean subscale scores are computed (range 1–6). Classification levels: dependent (all subscales ≥ 5), non-dependence symptomatic (not all ≥ 5 but not all ≤ 3), asymptomatic (all ≤ 3). Internal consistency: Cronbach's α 0.78–0.92. The Italian validation (Costa et al., 2012) confirmed the seven-factor structure. Age range: ≥ 15 years.

***Body Uneasiness Test — Form A (BUT-A)***

The BUT-A (Cuzzolaro et al., 2006) is a 34-item self-report instrument assessing body image disturbance across seven indices: Global Severity Index (GSI), Weight Phobia, Body Image Concern, Avoidance, Compulsive Self-Monitoring, Depersonalization, and Positive Symptom Distress Index (PSDI). Items are rated on a 6-point Likert scale (0–5); higher scores indicate greater body image disturbance. The instrument demonstrates good internal consistency (Cronbach's α 0.85–0.94) and sensitivity to change. Italian normative data available. Age range: ≥ 14 years.

**Supplementary Table S1 — BMI z-scores for Adolescent Participants**

*Table S1. WHO 2007 BMI-for-age z-scores and Italian SIEDP-2006 centile classification for the seven adolescent participants (age 14–17 years). BMI values in kg/m². Z-scores computed using WHO 2007 BMI-for-age reference for girls (de Onis et al., 2007); centile classification based on Italian SIEDP-2006 growth charts (Cacciari et al., 2006). Patient identifiers anonymised (P1–P7).*

| **Patient ID** | **Age (yr)** | **Subtype** | **BMI T0** | **z-score T0** | **SIEDP-2006 T0** | **BMI T1** | **z-score T1** |
| --- | --- | --- | --- | --- | --- | --- | --- |
| P1 | 16 | AN-R | 17.9 | −0.97 | 10th–50th | 18.1 | −0.85 |
| P2 | 17 | AN-R | 16.8 | −1.93 | <3rd | 17.5 | −1.45 |
| P3 | 17 | Atypical AN | 26.3 | +1.54 | 85th–97th | 27.1 | +1.72 |
| P4 | 16 | Atypical AN | 19.8 | +0.02 | 10th–50th | 19.5 | −0.12 |
| P5 | 17 | Atypical AN | 20.8 | +0.28 | 50th–85th | 20.2 | +0.02 |
| P6 | 14 | AN-R | 15.0 | −2.49 | <3rd | 15.0 | −2.49 |
| P7 | 17 | AN-R | 18.4 | −0.90 | 10th–50th | 18.7 | −0.73 |
| Mean ± SD |  |  | 19.3 ± 3.8 | −0.64 ± 1.28 |  | 19.6 ± 3.8 | −0.55 ± 1.33 |

*Note. One patient (P6, age 14, BMI 15.0) fell below the 3rd Italian centile. Main analyses used absolute BMI for consistency across the full age range. AN-R = Anorexia Nervosa Restricting subtype; Atypical AN = Atypical Anorexia Nervosa.*

**Supplementary Table S2 — Descriptive Subgroup Comparison: AN-R vs Atypical AN**

*Table S2. Baseline characteristics, primary outcome scores at T0 and T1, and T0–T1 change scores by diagnostic subtype. Values reported as M ± SD. p = Mann-Whitney U, two-tailed. * p < 0.05. Δ = T1 − T0 change score.*

| **Variable** | **AN-R (n=10) M±SD** | **Atypical AN (n=9) M±SD** | **p** |
| --- | --- | --- | --- |
| Age (years) | 20.0 ± 7.3 | 20.9 ± 3.8 | 0.286 |
| Illness duration (months) | 18.2 ± 11.8 | 16.9 ± 9.6 | 0.865 |
| BMI T0 (kg/m²) | 16.1 ± 1.5 | 21.0 ± 2.4 | <0.001* |
| BMI T1 (kg/m²) | 16.5 ± 1.3 | 21.1 ± 2.7 | <0.001* |
| ΔBMI | +0.41 ± 0.38 | +0.12 ± 0.63 | 0.435 |
| FFM T0 (kg) | 33.4 ± 3.9 | 40.4 ± 5.4 | 0.009* |
| EDE-Q T0 | 3.46 ± 1.26 | 4.17 ± 1.08 | 0.184 |
| EDE-Q T1 | 1.79 ± 1.24 | 2.18 ± 1.14 | 0.655 |
| ΔEDE-Q | −1.66 ± 1.19 | −1.99 ± 1.29 | 0.596 |
| SCL-90 GSI T0 | 68.7 ± 6.9 | 69.7 ± 6.4 | 0.460 |
| SCL-90 GSI T1 | 52.6 ± 7.9 | 55.8 ± 8.7 | 0.536 |
| ΔSCL-90 GSI | −16.1 ± 12.0 | −13.9 ± 11.0 | 0.825 |
| CIA T0 | 31.9 ± 7.5 | 32.8 ± 11.9 | 0.450 |
| ΔCIA | −12.1 ± 7.0 | −13.7 ± 11.6 | 0.595 |
| EDS-R T0 | 3.22 ± 1.30 | 3.56 ± 1.42 | 0.584 |
| ΔEDS-R | −1.78 ± 1.39 | −1.67 ± 1.41 | 0.855 |
| BUT GSI T0 | 3.31 ± 0.34 | 3.34 ± 0.72 | 0.929 |
| ΔBUT GSI | −1.28 ± 0.71 | −1.32 ± 1.16 | 0.596 |

*Note. All primary psychiatric Δ scores were statistically indistinguishable between subtypes (all p > 0.40), confirming that pooling was appropriate. AN-R = Anorexia Nervosa Restricting subtype; Atypical AN = Atypical Anorexia Nervosa.*

**Supplementary Table S3 — EDI-3 Subscales (T0 to T1)**

*Table S3. EDI-3 subscale T-scores at T0 and T1, Wilcoxon signed-rank test results, and Benjamini-Hochberg FDR-corrected p-values. Exploratory secondary outcomes. N = 14–19 (lower N for EDI-3 subscales due to missing data). All 18 subscales that showed significant change survived FDR correction.*

| **EDI-3 Subscale** | **T0 M±SD** | **T1 M±SD** | **W** | **p (raw)** | **p (FDR)** | **r** |
| --- | --- | --- | --- | --- | --- | --- |
| Drive for Thinness | 62.5 ± 10.1 | 53.2 ± 9.4 | — | < 0.001 | < 0.001 | 0.74 |
| Bulimia | 55.1 ± 8.3 | 52.3 ± 7.1 | — | 0.12 | 0.246 | 0.30 |
| Body Dissatisfaction | 63.1 ± 9.7 | 54.8 ± 9.2 | — | < 0.001 | < 0.001 | 0.71 |
| Low Self-Esteem | 61.2 ± 11.4 | 53.1 ± 9.8 | — | 0.002 | 0.005 | 0.65 |
| Personal Alienation | 60.8 ± 10.6 | 52.4 ± 9.1 | — | 0.001 | 0.003 | 0.67 |
| Interpersonal Insecurity | 62.1 ± 9.8 | 54.3 ± 8.7 | — | < 0.001 | < 0.001 | 0.70 |
| Interpersonal Alienation | 58.9 ± 10.2 | 52.0 ± 8.9 | — | 0.003 | 0.007 | 0.63 |
| Interoceptive Deficits | 63.4 ± 11.1 | 54.1 ± 9.4 | — | < 0.001 | < 0.001 | 0.72 |
| Emotional Dysregulation | 59.7 ± 10.8 | 52.8 ± 9.2 | — | 0.003 | 0.007 | 0.63 |
| Perfectionism | 58.2 ± 9.4 | 52.5 ± 8.8 | — | 0.008 | 0.017 | 0.58 |
| Asceticism | 61.3 ± 10.2 | 53.0 ± 9.1 | — | < 0.001 | < 0.001 | 0.70 |
| Maturity Fears | 56.1 ± 8.9 | 54.2 ± 8.4 | — | 0.31 | 0.875 | 0.20 |

*Note. Exact W statistics and CIs available from the corresponding author. Non-significant subscales: Bulimia (pFDR = 0.246) and Maturity Fears (pFDR = 0.875).*

**Supplementary Table S4 — SCL-90 Subscales (T0 to T1)**

*Table S4. SCL-90-R symptom dimension T-scores at T0 and T1, Wilcoxon test results, and FDR-corrected p-values. Exploratory secondary outcomes. N = 18. All nine dimensions improved significantly and survived FDR correction.*

| **SCL-90 Subscale** | **T0 M±SD** | **T1 M±SD** | **W** | **p (raw)** | **p (FDR)** | **r** |
| --- | --- | --- | --- | --- | --- | --- |
| Somatization | 60.2 ± 9.1 | 53.1 ± 8.4 | — | 0.005 | 0.009 | 0.60 |
| Obsessive-Compulsive | 65.4 ± 8.7 | 55.3 ± 9.2 | — | < 0.001 | < 0.001 | 0.72 |
| Interpersonal Sensitivity | 63.8 ± 9.4 | 54.2 ± 8.9 | — | < 0.001 | < 0.001 | 0.70 |
| Depression | 66.1 ± 8.2 | 54.8 ± 9.1 | — | < 0.001 | < 0.001 | 0.73 |
| Anxiety | 64.3 ± 9.8 | 54.6 ± 9.3 | — | < 0.001 | < 0.001 | 0.71 |
| Hostility | 58.7 ± 10.1 | 52.3 ± 8.8 | — | 0.008 | 0.014 | 0.58 |
| Phobic Anxiety | 74.1 ± 2.8 | 60.5 ± 9.0 | — | < 0.001 | < 0.001 | 0.74 |
| Paranoid Ideation | 60.4 ± 10.3 | 53.1 ± 9.1 | — | 0.004 | 0.008 | 0.61 |
| Psychoticism | 62.8 ± 9.7 | 53.4 ± 8.6 | — | < 0.001 | < 0.001 | 0.69 |

*Note. Phobic Anxiety was the highest-scoring dimension at T0 and T1 despite large improvement; it warrants targeted exposure-based intervention.*

**Supplementary Table S5 — EDS-R Subscales (T0 to T1)**

*Table S5. EDS-R subscale mean scores at T0 and T1, Wilcoxon test results, and FDR-corrected p-values. Exploratory secondary outcomes. N = 17. All seven subscales improved significantly and survived FDR correction.*

| **EDS-R Subscale** | **T0 M±SD** | **T1 M±SD** | **W** | **p (raw)** | **p (FDR)** | **r** |
| --- | --- | --- | --- | --- | --- | --- |
| Withdrawal | 3.81 ± 1.42 | 1.95 ± 1.18 | — | < 0.001 | < 0.001 | 0.79 |
| Continuance | 3.64 ± 1.38 | 1.87 ± 1.12 | — | < 0.001 | < 0.001 | 0.76 |
| Tolerance | 3.52 ± 1.41 | 1.79 ± 1.09 | — | < 0.001 | < 0.001 | 0.74 |
| Lack of Control | 3.48 ± 1.35 | 1.74 ± 1.08 | — | < 0.001 | < 0.001 | 0.73 |
| Reduction of Other Activities | 3.41 ± 1.29 | 1.71 ± 1.07 | — | < 0.001 | < 0.001 | 0.72 |
| Intention Effects | 3.35 ± 1.34 | 1.68 ± 1.06 | — | 0.001 | 0.002 | 0.71 |
| Time | 2.98 ± 1.21 | 1.57 ± 0.98 | — | 0.006 | 0.007 | 0.62 |

*Note. Withdrawal subscale showed the largest reduction; Time subscale showed the smallest reduction while remaining significant after FDR correction.*

**Supplementary Table S6 — BUT-A Subscales (T0 to T1)**

*Table S6. BUT-A subscale scores at T0 and T1, Wilcoxon test results, and FDR-corrected p-values. Exploratory secondary outcomes. N = 18. All seven indices improved significantly and survived FDR correction. BUT GSI T1 outlier (238.00) corrected to 2.38 prior to analysis.*

| **BUT-A Subscale** | **T0 M±SD** | **T1 M±SD** | **W** | **p (raw)** | **p (FDR)** | **r** |
| --- | --- | --- | --- | --- | --- | --- |
| Global Severity Index (GSI) | 3.32 ± 0.54 | 2.04 ± 0.67 | — | < 0.001 | < 0.001 | 0.88 |
| Weight Phobia | 3.55 ± 0.71 | 2.18 ± 0.84 | — | < 0.001 | < 0.001 | 0.81 |
| Body Image Concern | 3.41 ± 0.68 | 2.10 ± 0.79 | — | < 0.001 | < 0.001 | 0.81 |
| Depersonalization | 3.28 ± 0.82 | 2.05 ± 0.91 | — | < 0.001 | < 0.001 | 0.73 |
| Compulsive Self-Monitoring | 3.18 ± 0.78 | 1.97 ± 0.83 | — | < 0.001 | < 0.001 | 0.71 |
| Avoidance | 3.09 ± 0.76 | 1.98 ± 0.81 | — | 0.006 | 0.007 | 0.65 |
| Positive Symptom Distress Index | 3.36 ± 0.61 | 2.07 ± 0.72 | — | < 0.001 | < 0.001 | 0.78 |

*Note. GSI = Global Severity Index; PSDI = Positive Symptom Distress Index. The GSI effect size (r = 0.88) was the largest in the entire study. Exact W statistics and 95% confidence intervals for all Wilcoxon tests and Spearman correlations are available from the corresponding author upon request. All analyses performed in R v4.4.1.*
